# A Longitudinal Mediation Analysis of the Interrelations among Exclusionary Immigration Policy, Ethnic Identity, and Self-Esteem of Latinx Early Adolescents

**DOI:** 10.1007/s10964-023-01801-x

**Published:** 2023-06-16

**Authors:** Madonna P. Cadiz, Carlos E. Santos, Tristan D. Tibbe

**Affiliations:** 1 Department of Social Welfare, University of California, Los Angeles, Los Angeles, CA USA; 2 Department of Psychology, University of California, Los Angeles, Los Angeles, CA USA

**Keywords:** Discrimination, Immigration policy, Early adolescent, Latinx/Latina/Latino, Self-esteem, Ethnic identity

## Abstract

Little is known about how exclusionary immigration laws affect ethnic identity and self-esteem among Latinx middle school students. Arizona’s SB 1070, which required local officers to verify the legal status of detained individuals, garnered national attention for its impact on immigrant and Latinx communities. This study tested a longitudinal parallel multiple mediation model where perceptions of the effects of an exclusionary immigration law (Arizona’s SB 1070) on self-esteem were mediated by dimensions of ethnic identity (ethnic centrality, ethnic private regard, ethnic public regard). Data were collected from a two-wave survey of 891 early adolescents ranging in age from 10 to 14 years (*M* = 12.09 years; SD = 0.99), a majority (71%) of whom were of Mexican descent. Analyses revealed an indirect effect of T1 perceptions of this law on T2 self-esteem (7 months later), holding T1 measures constant, with T2 ethnic centrality, private regard, and public regard acting as mediators. Perceived effects of this exclusionary law led to increased self-esteem through increased dimensions of ethnic identity. Results reveal how ethnic identity functions as a multidimensional construct in the process through which exclusionary immigration policy may impact the self-esteem of Latinx early adolescents.

## Introduction

Research with non-White adolescents indicates exposure to racism and xenophobia can trigger the exploration of ethnic identity (Umaña-Taylor et al., 2014), which can lead to the development of positive affect related to one’s ethnic group (Quintana, 2007). With few exceptions, youth of color with positive ethnic identity demonstrate fewer indicators of negative adjustment (e.g., depressive symptoms, problem behaviors) and better positive adjustment (e.g., positive social functioning and well-being; Rivas-Drake et al., 2014) compared to peers with neutral or negative attitudes regarding their ethnic identity. Although the research literature on ethnic identity development among Latinx youth is vast, critical gaps exist in studies of relations between macro-level forms of discrimination, such as state-level policies affecting immigrant communities, and Latinx youth’s ethnic identity and self-esteem. Considering the ever-present tensions in national immigration policy debates—particularly in relation to Latinx populations—studies on the direct and indirect effects of exclusionary immigration policies on individual well-being among Latinx youth may help illuminate important avenues for intervention. The present study explores the interrelations and potential causal relations among exclusionary immigration policy, ethnic identity, and self-esteem in a sample of Latinx early adolescents.

### Theoretical Framework

The present study is informed by the rejection-identification model (RIM), which was originally developed by Branscombe et al. (1999) and draws from social identity theory (SIT; Tajfel & Turner, 1986). The RIM posits that exposure to ethnic discrimination by dominant groups can foster increased involvement in and a stronger sense of belonging with one’s own ethnic group, which in turn may promote positive psychological well-being. According to SIT and the corresponding self-esteem hypothesis (Martiny & Rubin, 2016), establishing oneself as a member of a particular social group creates a social identity that is fundamental to one’s sense of self, which increases the motivation to promote the status of one’s social group in larger society to maintain or improve one’s self-esteem. This process, while seemingly counter-intuitive in that exposure to discrimination results in improved well-being, can occur when individuals feel they are being rejected by the dominant group in a society. In response to such rejection, individuals may align themselves with a group to which they do belong, which results in an increased sense of belonging and improvements in self-esteem.

Investigators tested the RIM with Latinx early adolescents by investigating associations between two types of perceived discrimination—individual-level (i.e., direct, personal experiences) and group-level (i.e., perceptions attributed to a whole group)—and the outcome of personal self-esteem, with ethnic identity acting as a mediator (Armenta & Hunt, 2009). Results indicated perceived group discrimination was associated with higher self-esteem, both directly and indirectly, through increased ethnic identity. In contrast, perceived individual discrimination was related to lowered self-esteem through both direct and indirect (i.e., mediated by decreased ethnic identity) routes. Conversely, a longitudinal study (Cronin et al., 2012) found perceived individual discrimination during Latinx participants’ first year of college was indirectly associated with increased self-esteem 3 years later, with higher ethnic identity as the mediating variable. These extant studies indicate the relationship between perceived discrimination and self-esteem through ethnic identity remains unclear.

### Exclusionary State Policy toward Immigrants

Historically, the US has wavered in its attitude toward immigration from Latin American regions. Inclusive policies like the Hart-Cellar Act of 1965 have been negated or limited by restrictive policies, like the Immigration Reform and Control Act of 1986. Such policies can shape and are shaped by national public sentiments regarding immigrants, resulting in a *policy feedback loop* (Massey & Pren, 2012). For instance, the termination of the Bracero Program and implementation of the Hart-Cellar Act soon afterward coincided with a dramatic rise in undocumented immigration, as the demand for immigrant labor did not decrease once restrictive policies were implemented. As a result, employers were forced to break the law when hiring undocumented workers. This phenomenon contributed to the ‘Latino threat narrative’, which continues to pervade national political discourse around immigrants in the U.S.

#### Senate bill 1070

Arizona was a site of hostile anti-immigrant sentiment in 2010, when Senate Bill 1070 (SB 1070) was passed (Santos et al., 2013). This bill mandated that local police verify the immigration status of any person whom they have detained as part of a lawful stop, detention, or arrest, particularly if there was any suspicion that the individual was residing in the country without proper documentation. SB 1070 also made it illegal for undocumented individuals to enter private or public lands or to seek work in public areas without proof of legal residence. Altogether, SB 1070 was the culmination of a decade-long effort in Arizona to make the state as inhospitable as possible for immigrants and anyone perceived to be foreign-born, regardless of their actual citizenship status. Indeed, by 2014 the state was ranked by political scientists as the most hostile anti-immigrant state in the country (Pham & Van, 2014).

#### Exclusionary immigration policy as ethnic discrimination

Ethnic discrimination refers to behaviors and attitudes that serve to exclude, devalue, or treat unfairly those with an ethnic identity that distinguishes them from the majority group in a given society (Wong et al., 2003). Ethnic discrimination can be perpetrated by individuals within the micro- (e.g., family, close friends), meso- (e.g., neighborhood, school), and macro-level systems (e.g., government, society; Bronfenbrenner, 1977). In a study of the effects of different types of discrimination on adolescents, results showed significant differences in the types of ethnic discrimination perpetrated by peers (i.e., micro-level), school personnel (i.e., meso-level), and public-facing individuals like police officers and store owners (i.e., macro-level; Benner & Graham, 2013). Depending on the source of discrimination, ethnic discrimination had differential effects on adolescents. Similarly, SB 1070 gave police officers the power to enact macro-level ethnic discrimination at the individual level by directly apprehending people they considered suspicious.

#### Effects of immigration policy on well-being among Latinx individuals

Exclusionary immigration policies may have deleterious effects on various biopsychosocial indicators among Latinx individuals in the US. In an overarching review, associations were highlighted between exclusionary policies and various health-related outcomes, such as lower rates of health care utilization, lower ratings of overall health and mental health, and increased reports of perceived discrimination and acculturative stress (Perreira & Pedroza, 2019). Several studies identified significant associations between national or state-level anti-immigrant policies and indicators of poor psychological well-being, including: increased anxiety (Eskenazi et al., 2019); higher frequency of days with poor mental health (Hatzenbuehler et al., 2017); worsening stress levels (Philbin et al., 2018); and elevated levels of psychological distress (Szkupinski Quiroga et al., 2014). In a study of anti-immigrant policy and ethnic identity among Latinx middle-school students (Roehling et al., 2010), results showed youth surveyed during the peak of national immigration debates were more likely to begin exploring their ethnic identity, compared to a previous same-age cohort that was not acutely exposed to such debates. These findings lend support to the argument that ethnic identity exploration arises after exposure to racism and xenophobia (Quintana, 2007).

### Multidimensional Models of Ethnic Identity

There are two primary approaches to measuring and defining ethnic identity as a multidimensional construct (Rivas-Drake et al., 2014). One is concerned with ethnic identity *process*, including, for example, the process of exploring one’s membership in an ethnic group (Phinney, 1992). In contrast, the present study focuses on ethnic identity *content*. The multidimensional model of racial identity (MMRI; Sellers et al., 1998), based on a large body of research exploring racial identity development among African Americans in the U.S., proposes that racial identity consists of multiple components, including: racial centrality, racial public regard, and racial private regard. Studies of ethnic identity content and its effects on various outcomes among Latinx youth (e.g., Gillen-O’Neel et al., 2011; Hughes et al., 2011) incorporate these dimensions using the following terms: *ethnic centrality*, which denotes how central ethnic identity is to one’s overall sense of self and is considered a relatively stable trait over the life course once established; *ethnic private regard*, which represents affect regarding one’s membership in an ethnic group and is often measured as a positive dimension (e.g., pride); and *ethnic public regard*, which signifies beliefs about how one’s ethnic group is perceived by the larger societal context in which one inhabits. Recognition of ethnic identity as a multidimensional construct allows for the exploration of the specific effects of individual components of ethnic identity, as well as acknowledges the complexity of a construct such as ethnic identity.

Early adolescence is a period of rapid social, cognitive, emotional and physiological changes, which is marked by greater engagement with various social processes, including the meaning(s) of identity to oneself and to the social, cultural, and political contexts in which adolescents are embedded. Ethnic identity may be particularly salient for minoritized youth in middle school, as youth at this developmental stage become much more aware of the identity and public perceptions of groups to which they belong compared to younger children (Umaña-Taylor et al., 2014). Specifically, group-esteem, or positive feelings about being a member of an ethnic group, was found to increase among Latinx early and middle adolescents (French et al., 2006). A longitudinal study of students from sixth to ninth grade also showed a pattern of increasing ethnic behaviors and sense of belonging among Latinx adolescents (Huang & Stormshak, 2011). Consequently, aspects of ethnic identity are significantly related to important outcomes among Latinx youth, including: associations between ethnic public regard and academic achievement (Rivas-Drake, 2011) and college-going self-efficacy (Gonzalez et al., 2013); ethnic private regard and GPA among Latinx male adolescents (Umaña-Taylor et al., 2012); and ethnic private regard and well-being, symptoms of depression or anxiety, and engagement in risky behaviors (Rivas-Drake et al., 2014). These studies indicate how ethnic identity as a multidimensional construct becomes a prominent aspect of Latinx youth’s lives as they enter adolescence.

### Ethnic Identity and Self-Esteem among Latinx Early Adolescents

Self-esteem is a key indicator of well-being in early adolescence, a critical stage that influences later development. Lower self-esteem among early adolescents has been associated with engagement in risky behaviors (McClure et al., 2010) and other health-compromising behaviors (McGee & Williams, 2000), including substance use and abuse (Oshri et al., 2017), poor mental health (for a review, see Sowislo & Orth, 2013), and bleak economic prospects in adulthood (Trzesniewski et al., 2006). Similarly, research shows ethnic identity and self-esteem are interrelated among Latinx youth (Bracey et al., 2004; Quintana, 2007), with ethnic identity acting as a protective factor and possibly even boosting self-esteem among young Latinx adolescents (Neblett et al., 2012). This is potentially due to increased access to social and instrumental support in their ethnic and/or cultural communities (Phinney et al., 2001; Umaña-Taylor & Updegraff, 2007).

#### Ethnic centrality and self-esteem

Studies of the effects of ethnic centrality on self-esteem among Latinx youth indicate conflicting results. Studies with Latinx college-age students found significant indirect effects of ethnic cultural socialization (Rivas-Drake, 2011) and perceived group discrimination (Spencer-Rodgers & Collins, 2006) on positive self-esteem through ethnic centrality. In contrast, multivariate analyses of data collected from Asian, Latinx, and European American students found that perceived discrimination instigated by both adults and peers was a significant predictor of low self-esteem, and this effect was not moderated by ethnic centrality (Huynh & Fuligni, 2010). Considering the mixed results from studies of ethnic centrality and self-esteem among Latinx youth, further investigations of the relationship between these variables are warranted.

#### Ethnic private regard and self-esteem

Studies of ethnic private regard, or positive affect regarding one’s ethnic identity, among Latinx youth have consistently found significant associations between ethnic private regard and positive adjustment (Rivas-Drake et al., 2014). Latinx youth’s positive feelings toward their ethnic group were associated with: psychological well-being (Ghavami et al., 2011; Kiang et al., 2006), fewer symptoms of depression and anxiety (Berkel et al., 2010), and academic motivation (Fuligni et al., 2005; Perreira et al., 2010). Notably, two longitudinal studies (Kaplan et al., 2009; Umaña‐Taylor et al. (2009) found the positive effects of ethnic private regard on self-esteem among Latinx adolescents to be consistent over time.

#### Ethnic public regard and self-esteem

Among the ethnic identity dimensions of interest, ethnic public regard is perhaps the least represented in the ethnic identity literature. One study of ethnic-racially diverse university students—including Latinx youth—found a statistically significant effect on positive self-esteem when youth perceived members of their own ethnic group to exhibit positive ethnic public regard (Perkins et al., 2014). Another study of Latinx university students indicated that positive ethnic public regard had a significant effect on positive self-esteem, with increased sense of community acting as a mediator. The few studies of ethnic public regard among Latinx youth indicate this dimension may have a significant effect on positive self-esteem (Rivas-Drake, 2012).

## Current Study

Limited knowledge exists on the longitudinal associations among anti-immigrant state policy, youth ethnic identity, and youth self-esteem. The aim of this study is to analyze the impact of exclusionary immigration laws, such as SB 1070, on Latinx youth, as well as how such laws may influence youth self-esteem via youth ethnic identity. The following hypotheses guide this study: increases in Latinx youth’s perceptions of SB 1070’s impact on their lives will result in significant decreases in youth’s ethnic centrality, ethnic private regard, and ethnic public regard (Hypothesis 1); all three ethnic identity dimensions in this study (i.e., ethnic centrality, ethnic private regard, and ethnic public regard) will have significant positive effects on self-esteem among Latinx youth (Hypothesis 2); and the combined associations among predictor (T1 affected by SB 1070), mediating (T2 ethnic centrality, T2 ethnic private regard, and T2 ethnic public regard), and outcome (T2 self-esteem) variables will result in statistically significant indirect effects (Hypothesis 3). The longitudinal design of this study’s model, along with the unique measurement of ethnic discrimination via an immigration policy’s impact on individuals and the longitudinal aspect of the model, adds a novel aspect to and potentially extend the theoretical framework of the RIM.

## Methods

### Participants

Study participants included early adolescents attending an urban middle school in the state of Arizona, where 31% of the population identifies as Hispanic or Latino, and of these, 89% are Mexican (Ahn et al., 2022). Data were collected during a period of social and political unrest in this community, with policies targeting immigrant families looming large in political debates. Participants ranged in age from 10 to 14 years (*M* = 12.10 years; SD = 0.97), and slightly over half (52%) were female. Latinx youth accounted for a majority of the student population in the school, while most teachers and school staff were White. Most of the students (90%) qualified for free or reduced lunch. Like other low-income Latinx Arizonans—who reported the third lowest median income of any racial or ethnic group in the state (Ahn et al., 2022)—participants and their families were considered economically disadvantaged. This study focused on Latinx-identifying students, a large percentage of whom were of Mexican descent (84%), with the remaining indicating they were of Puerto Rican descent (2%) or other Hispanic, Latino, or Spanish origin (14%). Over half (56%) of participants were U.S.-born with at least one foreign-born parent (“second generation”), whereas 30% were U.S.-born with both parents also U.S.-born (“3+ generation”), and 14% were foreign-born with foreign-born parents (“first generation”). This is consistent with youth in Arizona overall, who tend to be U.S.-born (Ahn et al., 2022). The final sample for the present study consisted of 891 adolescents of Latinx origin.

### Procedure

Prior to participation in this study, all eligible middle school students and their parents received passive consent forms detailing the aims of the study, including an option to refuse to participate. To accommodate parents of varied monolingual or bilingual levels, consent forms were provided in both Spanish and English. Students also read and completed assent forms before being given the survey. Surveys were administered at three time points: Wave 1 occurred in February 2011 (T0), Wave 2 occurred in October 2011 (T1), and Wave 3 occurred in May 2012 (T2). The first wave did not include data for the focal predictor, but data from other variables collected during this wave were used to inform the missing data handling procedure (see Analytic Plan and supplementary material for more information). Research assistants administered the surveys during two social studies class periods. While one assistant read the survey aloud, one to three other assistants walked around the room to respond to students’ questions or provide clarification as needed. Since all participants understood and read English well, all surveys were administered in English. As a token of appreciation upon completion of the survey, each student received a rubber bracelet indicating the name of the project. All procedures involved in this study were reviewed by the university’s institutional review board before being approved.

### Measures

#### Affected by SB 1070

The focal predictor, *affected by SB 1070*, represents the average of four items (*α* = 0.92), which were created and previously published by the principal investigator to explore youth’s perceptions of how they were affected by the Arizona immigration law that passed in the state, SB 1070 (Santos et al., 2013). A team of researchers and content experts engaged in an iterative process to decide on item wording. Focusing on salient terms for middle-school age youth, the team developed the following survey items: “How affected are [you/your family/your community/your friends] by the recent act called SB 1070?”, and all were measured at T1. The response options for this item ranged from: 1 = *Not at all affected* to 9 = *Very affected*. A one-point increase on this measure reflects an increase in being affected by SB 1070. Results from exploratory (EFA) and confirmatory (CFA) factor analyses indicated a clear one-factor structure, with factor loadings ranging between 0.80–0.90 from the EFA and 0.84–0.92 from the CFA (see supplementary material for additional details).

#### Ethnic centrality

The first mediating variable of interest, *ethnic centrality*, was measured at both timepoints and represents the average of 5 items (*α* = 0.86), which were adapted from the Multidimensional Inventory of Black Identity (MIBI; Sellers et al., 1998) to different ethnic groups. Similar adaptations of this measure demonstrated acceptable validity (Gillen-O’Neel et al., 2011; Hughes et al., 2011; Johnson et al., 2005). Items include: “Overall, being a member of my ethnic group has a lot to do with how I feel about myself”; “Being a part of my ethnic group is an important reflection of who I am”; and “Being a part of my ethnic group is important for my social relationships”. Participants were given a range of response options on a 5-point Likert-type scale, from 1 = *Strongly disagree* to 5 = *Strongly agree*. Correspondingly, a one-point increase on this measure reflects increasing agreement with each item.

#### Ethnic private regard

The second mediating variable of interest, *ethnic private regard*, was measured at T1 and T2 and represents the average of 3 items (*α* = 0.89) that, like the items for the measure of ethnic centrality, were also adapted from the Multidimensional Inventory of Black Identity (Sellers et al., 1998). These items were worded as: “I am happy that I am a member of my ethnic group”; “I am proud to be a member of my ethnic group”; and “I feel good about the people of my ethnic group”. Response options were on a 4-point Likert-type scale, with 1 = *Almost never*, 2 = *Sometimes*, 3 *=* *Often*, and 4 = *Very often*. A one-point increase on this measure reflects increasing frequency of endorsement of each item.

#### Ethnic public regard

The third and final mediating variable, *ethnic public regard*, was measured at both timepoints and represents the average of 3 items (*α* = 0.81) adapted from the Multidimensional Inventory of Black Identity (Sellers et al., 1998). Phrasing for the 3 items were as follows: “Most people think that people of my ethnic group are as smart as people of other ethnic groups”; “People think that people of my ethnic group are as good as people from other ethnic groups”; and “People from other ethnic groups think that people of my ethnic group have made important contributions”. A 4-point Likert-type scale was used for participants to give their chosen response, with 1 = *Almost never*, 2 = *Sometimes*, 3 = *Often*, and 4 = *Very often*. A one-point increase on this measure reflects the participant’s increasing endorsement of each item.

#### Self-esteem

Rosenberg’s Global Self-Esteem Scale (*α* = 0.81; Hensley & Roberts, 1976) was used to assess self-esteem at both T1 and T2. Participants were instructed to rate how much they agreed with ten items, such as: “I certainly feel useless at times,” “I take a positive attitude toward myself,” and “I feel I do not have much to be proud of.” The items were rated on a 4-point Likert-type scale, from 1 = *Strongly disagree* to 4 = *Strongly agree*. A one-point increase on this measure corresponds to increasing agreement with each item.

#### Demographic Variables

Students’ self-reported gender was collected as a binary measure (1 = male). Immigrant generation was measured with three discrete options: child and both parents foreign-born (first generation), US-born child with at least one foreign-born parent (second generation), and child and both parents US-born (“3 + ” generation). Gender, immigrant generation, and age were included as covariates in post-hoc analyses (see sensitivity analyses and supplementary material).

### Analytic Plan

**Fig. 1:**
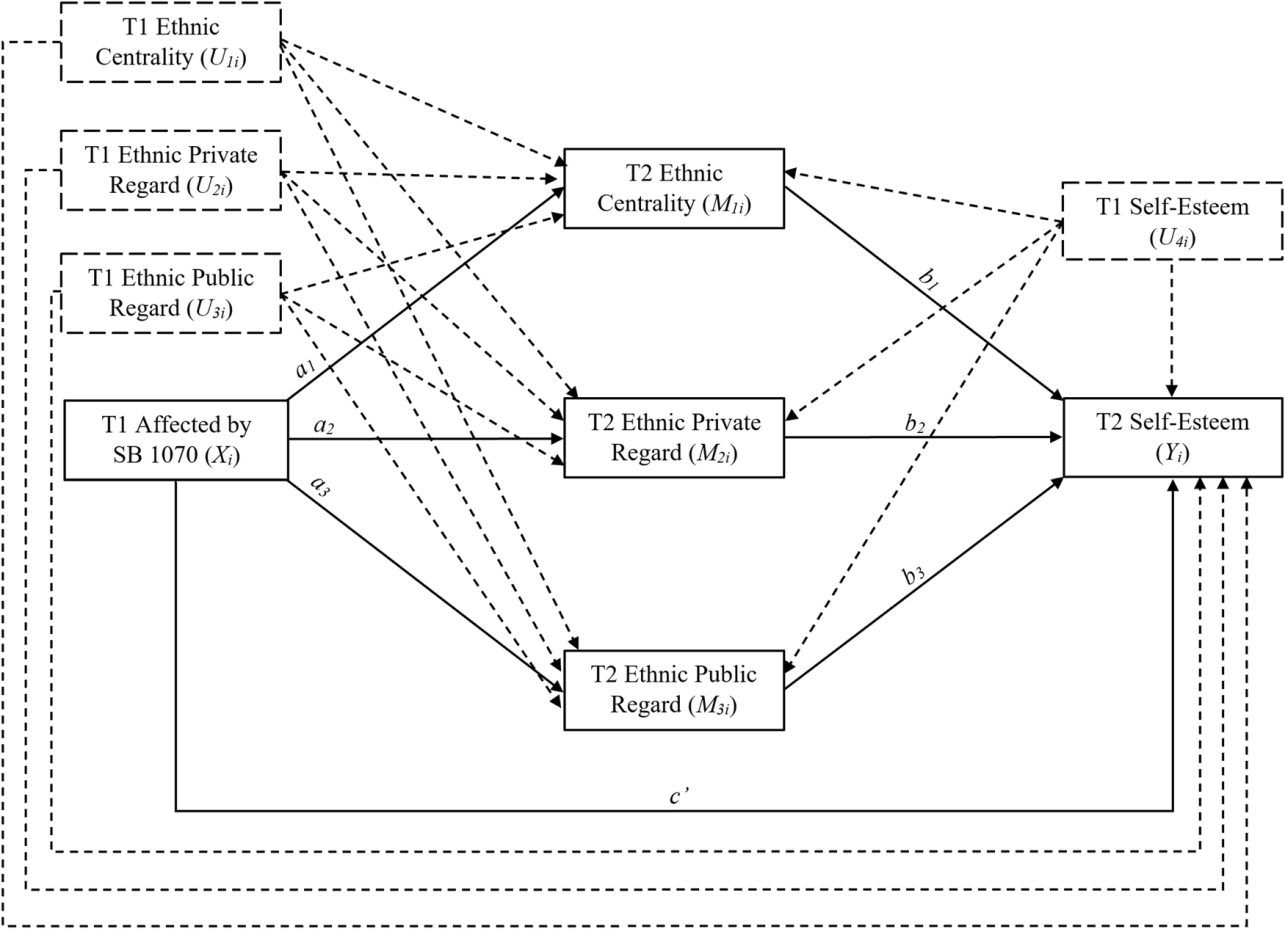
*Conceptual Diagram of Parallel Multiple Mediation Model*. A conceptual diagram of the proposed model with covariates that represents the direct and indirect effects of T1 affected by SB 1070 on T2 self-esteem through a multiple mediation process, whereby T1 affected by SB 1070 simultaneously predicts T2 ethnic centrality, T2 ethnic private regard, and T2 ethnic public regard, which together predict T2 self-esteem. T1 measures of ethnic centrality, ethnic private regard, ethnic public regard, and self-esteem are included as covariates in all equations

The multiple mediation model used in this study was conceptualized to assess if dimensions of ethnic identity—T2 ethnic centrality (i.e., mediating variable, *M*_1*i*_), T2 ethnic private regard (i.e., mediating variable, *M*_2*i*_), and T2 ethnic public regard (i.e., mediating variable, *M*_3*i*_)—act as mediators of the predictive relationship between early Latinx adolescents’ reports of how much they were affected by SB 1070 at T1 (i.e., focal predictor, *X*_*i*_) and overall self-esteem at T2 (i.e., dependent variable, *Y*_*i*_; see Fig. 1 for full conceptual diagram). To arrive at a more accurate assessment of the role of being affected by SB 1070 on later self-esteem, included in the model are the following covariates: T1 ethnic centrality (i.e., control variable, *U*_*1i*_), T1 ethnic private regard (i.e., control variable, *U*_*2i*_), T1 ethnic public regard (i.e., control variable, *U*_*3i*_), and T1 overall self-esteem (i.e., control variable, *U*_*4i*_), so as to control for the effects of repeated measurements. Covariance of repeated measures can significantly confound the interpretation of change in longitudinal mediation designs (MacKinnon et al., 2007); thus, inclusion of these T1 measures as covariates are likely to lead to more accurate estimates of effects.

The following equations are represented in Fig. 1: $\hat M_{1i}$ = *i*_*M1*_ + *a*_*1*_*X*_*i*_ + *u*_*1*_*U*_*1i*_ + *u*_*2*_*U*_*2i*_ + *u*_*3*_*U*_*3i*_ + *u*_*4*_*U*_*4i*_ (T1 affected by SB 1070 predicting T2 ethnic centrality); $\hat M_{2i}$ = *i*_*M2*_ + *a*_*2*_*X*_*i*_ + *u*_*1*_*U*_*1i*_ + *u*_*2*_*U*_*2i*_ + *u*_*3*_*U*_*3i*_ + *u*_*4*_*U*_*4i*_ (T1 affected by SB 1070 predicting T2 ethnic private regard); $\hat M_{3i}$ = *i*_*M3*_ + *a*_*3*_*X*_*i*_ + *u*_*1*_*U*_*1i*_ + *u*_*2*_*U*_*2i*_ + *u*_*3*_*U*_*3i*_ + *u*_*4*_*U*_*4i*_ (T1 affected by SB 1070 predicting T2 ethnic public regard); and, *Ŷ*_*i*_ = *i*_*Y*_ + *c’X*_*i*_ + *b*_*1*_*M*_1*i*_ + *b*_*2*_*M*_2*i*_ + *b*_*3*_*M*_3*i*_ + *u*_*1*_*U*_*1i*_ + *u*_*2*_*U*_*2i*_ + *u*_*3*_*U*_*3i*_ + *u*_*4*_*U*_*4i*_ (T1 affected by SB 1070 predicting T2 self-esteem via T2 ethnic centrality, T2 ethnic private regard, and T2 ethnic public regard as parallel multiple mediators). Also included in the analyses was a test of the total effect, as expressed by the following equation (not depicted in Fig. 1): *Ŷ*_*i*_ = *c*_*0*_ + *c*_*1*_*X*_*i*_ + *c*_*2*_*U*_*1i*_ + *c*_*3*_*U*_*2i*_ + *c*_*4*_*U*_*3i*_ + *c*_*5*_*U*_*4i*_. To control for the effects of repeated measures of the dependent variable and mediating variables over time, measures at T1 of self-esteem (*α* = 0.83), ethnic centrality (*α* = 0.83), ethnic private regard (*α* = 0.85), and ethnic public regard (*α* = 0.75) were included in the above equations as predictors of all mediating and outcome variables at T2. Each control variable was measured using the same items and scales as for the target variables.

To address missing data—with missingness percentages falling in the range of 28–38% (see Table 1) for all variables in the model—Blimp Version 3.0.52 (Keller & Enders, 2021) was used to conduct model-based multiple imputation (Enders, 2022). Measures of ethnic centrality, ethnic private regard, ethnic public regard, and self-esteem taken at Wave 1 (T0) were used as auxiliary variables to inform the imputation procedure. A burn-in period of 5000 iterations was used for the potential scale reduction factor (Gelman & Rubin, 1992) to have adequate iterations to drop below 1.05, indicating acceptable convergence, before an additional 10,000 iterations were run to make the imputations. A total of 100 imputed datasets were generated and saved. The mitml package version 0.4.1 (Grund et al., 2021) in R Version 4.0.2 (R Core Team, 2021) was then used to fit regression models and pool parameter estimates and their standard errors across imputed datasets according to Rubin’s (1987) Rules.

**Table 1:** Descriptive analyses and missing data percentages of target variables

Variable	*M*	SD	Missing data (%)
1. T1 Affected by SB 1070	4.10	2.71	34
2. T2 Ethnic Centrality	3.45	0.85	38
3. T2 Ethnic Private Regard	4.18	0.78	38
4. T2 Ethnic Public Regard	3.38	0.85	38
5. T2 Self-Esteem	2.99	0.53	38
6. T1 Ethnic Centrality	3.65	0.79	28
7. T1 Ethnic Private Regard	4.29	0.70	28
8. T1 Ethnic Public Regard	3.44	0.83	28
9. T1 Self-Esteem	2.96	0.51	29

As described in Wu and Jia (2013), to conduct inference on the specific and total indirect effects, 1000 bootstrap samples were drawn from each of the 100 imputed datasets. For each bootstrap sample in each imputed dataset, bootstrap estimates of all three specific indirect effects and the total indirect effect were calculated and saved. At the end of the procedure, all 100,000 bootstrap estimates were pooled for each indirect effect and used to form 95% percentile bootstrap confidence intervals. Additional information about the imputation/analysis procedures used in this study is available in the supplementary material.

## Results

Preliminary analyses of cases with complete data (*n* = 487) are found in Table 1 with descriptive statistics, and in Table 2 with bivariate correlations of all variables in the target model. On average, Latinx youth in this study reported being moderately negatively affected by SB 1070 (*M* = 4.10; SD = 2.71 on a scale ranging from 1 to 9). As expected, T1 and T2 variables of the same measures were highly correlated, with the strongest correlation between the T1 and T2 measures of self-esteem, *r*(251) = 0.56, *p* < 0.001. Both T1 and T2 measures of ethnic private regard, as well as the T1 measure of ethnic centrality, were significantly correlated with T1 affected by SB 1070. T2 ethnic centrality was also highly correlated with T2 ethnic private regard, *r*(258) = 0.43, *p* < 0.001 and T2 ethnic public regard, *r*(258) = 0.44, *p* < 0.001. This can be expected, considering the similarities inherent in these variables’ focus on ethnic identity. T2 self-esteem was significantly correlated with T2 ethnic private regard *r*(254) = 0.19, *p* = 0.002 and with T2 ethnic public regard, *r*(254) = 0.21, *p* = 0.001, indicating strong associations among these variables.

**Table 2:** Bivariate correlations of target variables

Variable	1	2	3	4	5	6	7	8
1. T1 Affected by SB 1070	–							
2. T2 Ethnic Centrality	0.11	–						
3. T2 Ethnic Private Regard	0.15*	0.43**	–					
4. T2 Ethnic Public Regard	−0.02	0.44**	0.30**	–				
5. T2 Self-Esteem	−0.09	0.06	0.19**	0.21**	–			
6. T1 Ethnic Centrality	0.18**	0.47**	0.33**	0.19**	0.10	–		
7. T1 Ethnic Private Regard	0.23**	0.27**	0.37**	0.08	0.16*	0.50**	–	
8. T1 Ethnic Public Regard	0.03	0.21**	0.11	0.28**	0.17**	0.43**	0.32**	–
9. T1 Self-Esteem	−0.12	0.08	0.16*	0.29**	0.56**	0.26**	0.25**	0.28**

*p < 0.05; **p < 0.01

### Mediation Analyses

**Fig. 2:**
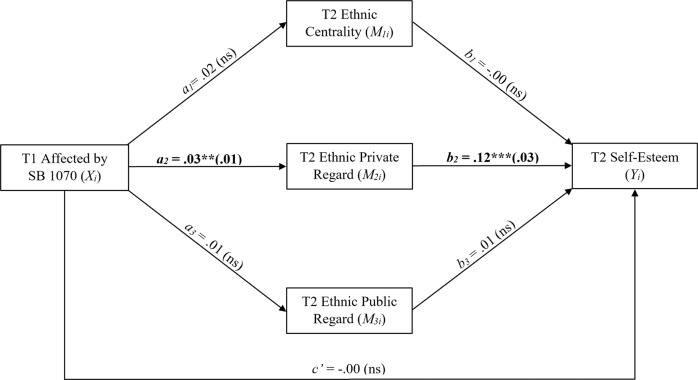
*Statistical Diagram of Results from Parallel Multiple Mediation Analysis*. A statistical diagram of the multiple mediation model that represents the following equations (not depicted but included in all equations are covariates T1 ethnic centrality, T1 ethnic private regard, T1 ethnic public regard, and T1 self-esteem): $\hat M_{1i}$ = 1.29 + 0.02*X*_*i*_ + 0.46*U*_*1i*_ + 0.10*U*_*2i*_ + 0.02*U*_*3i*_ – 0.05*U*_*4i*_ (T1 affected by SB 1070 predicting T2 ethnic centrality), $\hat M_{2i}$ = 1.46 + 0.03*X*_*i*_ + 0.11*U*_*1i*_ + 0.36*U*_*2i*_ + 0.03*U*_*3i*_ + 0.17*U*_*4i*_ (T1 affected by SB 1070 predicting T2 ethnic private regard), $\hat M_{3i}$ _=_ 1.30 + 0.01*X*_*i*_ + 0.07*U*_*1i*_ – 0.01*U*_*2i*_ + 0.31*U*_*3i*_ + 0.27*U*_*4i*_ (T1 affected by SB 1070 predicting T2 ethnic public regard_),_ and *Ŷ*_*i*_ = 0.95 – 0.00*X*_*i*_ – 0.00*M*_1*i*_ + 0.12*M*_2*i*_ + 0.01*M*_3*i*_ – 0.02*U*_*1i*_ – 0.03*U*_*2i*_ + 0.01*U*_*3i*_ + 0.57*U*_*4i*_ (T1 affected by SB 1070 predicting T2 self-esteem via T2 ethnic centrality, T2 ethnic private regard, and T2 ethnic public regard as multiple mediators). The numbers in parentheses indicate the standard error for each statistically significant coefficient. *p < 0.05. **p < 0.01. ***p < 0.001

Results from the parallel multiple mediation model are shown in Table 3 and Fig. 2. Of note, the second equation, representing the predictive relationship between T1 Affected by SB 1070 and T2 ethnic private regard, resulted in a statistically significant value of *a*_*2*_ = 0.03 (*t*(241) = 2.55, *p* = 0.01). That is, two middle schoolers who differ by one unit on T1 affected by SB 1070 are also expected to differ by 0.03 units on T2 ethnic private regard after controlling for T1 variables. The statistically significant value of *b*_*2*_ = 0.12 (*t*(175) = 3.79, *p* < 0.001) can be interpreted as: two youth who differ by one unit on T2 ethnic private regard are also expected to differ by 0.12 units on T2 self-esteem, holding constant T1 and other T2 variables.

**Table 3:** Results from regression analyses

Model	Coeff	SE	*t*	df	*p*
*Outcome: T2 Self-Esteem (Total Effect Model)*					
Intercept	1.137	0.160	7.104	228.442	<0.001
T1 Affected by SB 1070	0.000	0.007	−0.001	245.110	0.999
T1 Ethnic Centrality	−0.008	0.030	−0.279	246.669	0.781
T1 Ethnic Private Regard	0.013	0.035	0.360	205.608	0.720
T1 Ethnic Public Regard	0.021	0.026	0.796	263.682	0.427
T1 Self-Esteem	0.591	0.039	15.229	268.033	<0.001
*Outcome: T2 Ethnic Centrality*					
Intercept	1.286	0.263	4.882	254.463	<0.001
T1 Affected by SB 1070	0.019	0.012	1.487	256.650	0.138
T1 Ethnic Centrality	0.463	0.048	9.733	309.039	<0.001
T1 Ethnic Private Regard	0.104	0.053	1.956	307.427	0.051
T1 Ethnic Public Regard	0.022	0.044	0.490	260.604	0.624
T1 Self-Esteem	−0.048	0.066	−0.735	272.144	0.463
*Outcome: T2 Ethnic Private Regard*					
Intercept	1.461	0.291	5.021	183.819	<0.001
T1 Affected by SB 1070	0.032	0.013	2.553	240.629	0.011
T1 Ethnic Centrality	0.109	0.049	2.239	282.341	0.026
T1 Ethnic Private Regard	0.355	0.057	6.187	231.842	<0.001
T1 Ethnic Public Regard	0.034	0.044	0.775	252.301	0.439
T1 Self-Esteem	0.173	0.067	2.571	243.399	0.011
*Outcome: T2 Ethnic Public Regard*					
Intercept	1.303	0.283	4.596	249.107	<0.001
T1 Affected by SB 1070	0.005	0.014	0.376	218.867	0.707
T1 Ethnic Centrality	0.070	0.052	1.345	277.309	0.180
T1 Ethnic Private Regard	−0.015	0.058	−0.254	282.642	0.800
T1 Ethnic Public Regard	0.310	0.046	6.766	291.092	<0.001
T1 Self-Esteem	0.266	0.068	3.913	307.481	<0.001
*Outcome: T2 Self-Esteem*					
Intercept	0.952	0.168	5.678	217.535	<0.001
T2 Ethnic Centrality	0.001	0.030	0.017	187.838	0.987
T2 Ethnic Private Regard	0.117	0.031	3.788	175.303	<0.001
T2 Ethnic Public Regard	0.011	0.025	0.450	264.596	0.653
T1 Affected by SB 1070	−0.004	0.007	−0.518	243.345	0.605
T1 Ethnic Centrality	−0.022	0.032	−0.697	245.063	0.487
T1 Ethnic Private Regard	−0.029	0.035	−0.818	223.838	0.414
T1 Ethnic Public Regard	0.013	0.027	0.489	249.803	0.625
T1 Self-Esteem	0.568	0.038	14.917	289.959	<0.001

### Testing Indirect Effects

Specific indirect effects were tested through each mediating variable—T2 ethnic centrality, T2 ethnic private regard, and T2 ethnic public regard—holding T1 covariates constant (see Table 4). First, the indirect effect of T1 affected by SB 1070 on T2 self-esteem through T2 ethnic centrality was not significant, *a*_*1*_*b*_*1*_ = 0.000, 95% CI [−0.001, 0.001]. Second, the indirect effect of T1 affected by SB 1070 on T2 self-esteem through T2 ethnic private regard was significant, *a*_*2*_*b*_*2*_ = 0.004, 95% CI [0.001, 0.008]. Third, the indirect effect of T1 affected by SB 1070 on T2 self-esteem through T2 ethnic public regard was not significant, *a*_*3*_*b*_*3*_ = 0.000, 95% CI [−0.001, 0.001]. The total indirect effect of T1 affected by SB 1070 on T2 self-esteem through all three simultaneous T2 ethnic identity content measures, while controlling for T1 covariates, was significantly different from zero, Σ(*a*_*i*_ x *b*_*i*_) = 0.004, 95% CI [0.000, 0.008].

**Table 4:** Results from indirect effects testing

Indirect effect	Estimate	*LLCI*	*ULCI*
Mediator: T2 Ethnic Centrality (*a*_*1*_*b*_1_)	0.000	−0.001	0.001
Mediator: T2 Ethnic Private Regard (*a*_*2*_*b*_2_)	0.004	0.001	0.008
Mediator: T2 Ethnic Public Regard (*a*_*3*_*b*_3_)	0.000	−0.001	0.001
Total Indirect Effect [Σ(*a*_*i*_*b*_*i*_)]	0.004	0.000	0.008

### Sensitivity Analyses

Although the final analysis of this study used multiple imputation in Blimp combined with the imputation-then-bootstrap approach (Wu & Jia, 2013) to conduct inference on the indirect effects, other popular multiple imputation programs such as the Amelia (Honaker et al., 2011) and mice (van Buuren et al., 2011) R packages exist, and the bootstrap-then-imputation approach (Zhang & Wang, 2013) offers an alternative method for conducting inference on the indirect effect. To see if the results held regardless of the methods used, sensitivity analyses were run using all combinations of the Amelia and mice multiple imputation programs with the imputation-then-bootstrap and bootstrap-then-imputation procedures. Equations that included the T1 covariates were also varied to see if the choice of which equations contain which T1 covariates impacted the results. Finally, models that included the demographic variables of age, gender, and immigration generation as covariates were also fit, resulting in a total of 25 alternate models generated. In all models, the specific indirect effect of T1 affected by SB 1070 on T2 self-esteem through T2 ethnic private regard remained significant, which was consistent with results from the final model. However, estimates of the total indirect effect were not significantly different from zero in eight of 25 alternate models (see supplementary material for further details).

## Discussion

For Latinx early adolescents, having a strong, positive ethnic identity may play a protective role in limiting the negative effects of discrimination these youth experience in the larger US context, particularly in states like Arizona that are known for targeting immigrants and Latinx communities. Though discrimination is often studied in this context as interpersonal experiences (e.g., being called racial or xenophobic slurs), fewer studies look at the impacts of macro-level forms of discrimination, such as state-level anti-immigrant policy, on ethnic identity development and its ties to developmental outcomes like self-esteem among early adolescents. This study aimed to fill this gap by making connections among these factors through longitudinal mediation analyses. The RIM (Branscombe et al., 1999, Armenta & Hunt, 2009) was applied as a theoretical framework for this study to better understand the potential links between the impacts of macro-level forms of discrimination on individuals, ethnic identity content, and self-esteem in Latinx early adolescents. Specifically, this study tested the relations between Latinx youth’s self-reports of how strongly affected they were by SB 1070, dimensions of ethnic identity, and self-esteem. Ethnic identity was operationalized as three separate variables—ethnic centrality, ethnic private regard, and ethnic public regard—which draws from the multidimensional model of racial identity (Sellers et al., 1998). The longitudinal associations between Latinx early adolescents’ being affected by SB 1070, ethnic identity variables, and self-esteem were all tested simultaneously.

Study results showed evidence of a significant positive association between the impact of SB 1070 on youth and later ethnic private regard. That is, those youth who rated themselves as being strongly affected by SB 1070 at the beginning of the school year also showed increased ethnic private regard, or positive feelings, toward their ethnic group at the end of the school year, holding constant ethnic identity content and self-esteem at the beginning of the school year. This result contradicts previous findings indicating individual-level discrimination predicted decreases in ethnic identity content among Latinx youth (Armenta & Hunt, 2009). This discrepancy in results is due perhaps to differences in measurement, as measurement of individual-level discrimination in the previous study consisted of a single item indicating global experiences of discrimination. In this study, the source of discrimination was macro-level state policy, yet the effects of such a policy were intimately felt by youth, as expressed by the items on the affected by SB 1070 measure. The results do support the theoretical framework of the RIM (Branscombe et al., 1999), in that being confronted with ethnic discrimination led to more positive attitudes among youth toward their ethnic group.

A significant positive association was also found between ethnic private regard and self-esteem after controlling for T1 self-esteem and ethnic identity content. That is, at the end of the school year, youth with higher ethnic private regard also had higher self-esteem. This result lends support to the RIM (Branscombe et al., 1999) and aligns with Armenta and Hunt’s (2009) findings showing a positive association between ethnic identity content and self-esteem. This result supports previous studies demonstrating the positive effects of ethnic private regard and positive psychological adjustment (Rivas-Drake et al., 2014).

Evidence was also found for a nonzero specific indirect effect of being affected by SB 1070 on later self-esteem through ethnic private regard. This finding contradicts the results from Armenta and Hunt’s (2009) study showing a significant indirect effect of perceived individual discrimination on lower self-esteem through ethnic identity content. These differential findings may be accounted for by differences in individual-level discrimination measurement, as the previous study’s measure focused on perceptions of interpersonal discrimination. These results ultimately support the RIM (Branscombe et al., 1999). For the Latinx early adolescents in this study, the impact of SB 1070 on their lives at the beginning of the school year led to increased self-esteem at the end of the year through higher ethnic private regard. The total indirect effect of SB 1070’s impact on youth on later self-esteem—with ethnic centrality, ethnic private regard, and ethnic public regard acting simultaneously as mediators—was also statistically significant. SB 1070 was implemented precisely to signal to Latinx immigrants and their family members that they were being excluded from dominant society; yet this blatant form of ethnic discrimination had the unintended and converse effect of boosting ethnic identity among Latinx adolescents in this study, which contributed to improved self-esteem among these youth. These results demonstrate that, not only can the consequences of macro-level state policy be felt by Latinx early adolescents, but that a policy like SB 1070, though explicitly anti-immigrant, can also have a positive effect on youth’s self-esteem through its influence on positive ethnic identity content overall, and specifically through positive ethnic private regard.

The statistically significant effects found in this study held longitudinally, with measures of each mediating and outcome variable from previous waves used to account for the effects of repeated measurements. The longitudinal aspect of these analyses indicate that anti-immigrant policy can have long-term effects on youth’s perceptions of their ethnic group. The results of this study indicate that Latinx youth are not simply passive recipients of anti-immigrant sentiment but may instead react and develop a more positive attitude toward their ethnic group, despite being faced with blatant state-level discrimination.

There were no significant associations between being affected by SB 1070 and the other two mediating variables in the model—later ethnic centrality and ethnic public regard—or with these two variables and self-esteem. These results are noteworthy because one might intuitively predict that an explicitly anti-immigrant policy like SB 1070 would lead to a decrease in ethnic public regard, or perceptions of the wider societal attitudes toward one’s ethnic group among youth. For the population in this study, where no such effect was detected, it could be that anti-immigrant sentiment was already strong in their social context, so that this new legislation may have represented not a stark change but further confirmation of the state’s xenophobic attitudes toward their ethnic group. It is less clear why there were no significant associations between ethnic centrality and our other study variables. These results suggest the possibility that SB 1070’s impact on self-esteem among Latinx youth may not be explained solely by ethnic centrality or ethnic public regard. As shown by the results from testing the total indirect effect, these two variables may work in concert with ethnic private regard to bolster self-esteem in response to ethnic discrimination.

The results of this study lend support to the existing literature linking exclusionary immigration policies to Latinx youth’s psychosocial outcomes. Similar studies have found links between exclusionary immigration policies and mental health (Santos et al., 2013), academic adjustment (Santos et al., 2018), and civic engagement (Terriquez et al., 2020). This study’s findings build on extant knowledge by establishing longitudinal mediating associations among experiences of discrimination via exclusionary immigration policy, dimensions of ethnic identity content, and self-esteem. Though the predictive effects of both exclusionary immigration policy and ethnic identity content on psychological well-being on Latinx adolescents have been demonstrated separately, no previous studies have found evidence of a mediated effect of exclusionary immigration policy on self-esteem through ethnic identity content. The findings of this study weave a connective thread among these factors over time.

### Study Limitations

The effects found in this study, though statistically significant, were quite small, making the practical significance of the results potentially difficult to ascertain. Eight of the 25 models fit in the sensitivity analyses failed to find a significant total indirect effect (see supplementary material), making the significance of this effect more tenuous. Further testing of the total indirect effect may be beneficial to confirm that the results from this study were not merely due to the inferential procedures used. This study looked at a specific anti-immigrant state-level policy—SB 1070—in one state, with youth surveyed within one school with a predominantly Latinx population. In addition, the reliance on youth self-report to assess their perceptions of the effects of SB 1070 on themselves, their family members, friends, and community members, introduces potential sources of bias, as youth may not accurately assess the effects of SB 1070 on individuals within their social circle. Although the effects of macro-level discrimination are typically measured in larger geographic units (e.g., state, nation), the SB 1070 measure in this study captured the perception effects of macro-level exclusionary immigration policy at an individual level. Notably, it is also unclear whether our findings and results would generalize to other subgroups of Latinx youth. Our study reflects a sample of largely Mexican-origin, low income, second generation and beyond group of Latinx youth in Arizona. It is plausible that patterns may differ in other US regions or among other subgroups of Latinx individuals. Thus, results are limited to the context of Arizona, individual participants’ reactions to SB 1070, and the time in which this policy was enacted.

Despite limitations related to the societal context in which data for this study were collected, some elements that were present at the time of SB 1070 enactment remain alive and well in today’s political context. The current presidential administration, though purportedly less hostile than the previous administration regarding its approach to immigrant populations, continues to implement similar or tougher immigration policies (Robles, 2023). This study calls attention to the ways in which policies regarding immigrants affect the ethnic identity and psychological well-being of Latinx youth who are disproportionately immigrants themselves or come from immigrant families. Policy makers may ultimately play a role in shaping the ethnic identity of Latinx youth, and this is important to consider in state-level and national debates concerning immigration.

Analyses of the total effect of youth’s perceptions of SB 1070 on their personal self-esteem indicated that there was no statistically significant relationship between these variables. This precludes application of the causal steps approach (Baron & Kenny, 1986), in which a significant total effect of the predictor variable on the outcome variable must be shown before a mediating effect can be analyzed. Though perhaps seen as a limitation by traditional mediation scholars, another argument is that evidence of a significant total effect need not be a prerequisite for a mediation analysis, as it can severely limit the types of mediating relations that can be explored (Hayes, 2017). In lieu of the causal steps approach, an alternate method of mediation analyses (Hayes, 2017) focuses on the associations between the predictor and mediating variables, and between the mediating and outcome variables, to produce an indirect effect. The results of this study are a testament to these assertions, since the statistically significant aspects of the model were only revealed through the separate paths, as well as the specific and total indirect effects, of the predictor variable on the outcome variable.

The mediation model applied in this study, with its longitudinal design and use of the RIM theoretical framework, is meant to demonstrate causal effects among the variables of interest. Regardless, causal claims must be made with caution since the study’s non-experimental design makes it difficult to determine whether there is a true causal relationship among variables or if another external variable is driving the association. Though the effects of previous timepoints were held constant, there is always the possibility in an observational study that an unknown confounding variable is the true cause. Despite the use of longitudinal data, both mediating and outcome variables were measured at the same timepoint (T2), meaning the specific temporal order of these variables cannot be confirmed through our research design. Thus, even if there is a causal relation between any one of the mediators and the outcome, the way the data were collected cannot itself be used to support change in the mediators occurring before change in the outcome. Instead of study design, this study relies on theory rooted in previous research to justify the claim that the ethnic identity dimensions mediate the relationship between SB 1070 and self-esteem. Yet, because both research design and theory are needed to determine whether any effect is causal, a lack of experimental design and longitudinal data does not preclude the possibility of drawing useful inferences from data when those inferences are based on informed theory (Hayes and Rockwood (2020)). This research should serve as a springboard from which more targeted (experimental) studies can arise to further investigate the processes we describe.

### Implications and Future Directions

The results of this study, which demonstrate a relationship between macro-level exclusionary state policy and Latinx youth self-esteem through ethnic identity content, are novel and carry significant implications. Findings provide evidence that such a relationship can and does exist within certain populations and requires deeper analysis of the potential effects of exclusionary state policy on immigrant groups. The results also lend further support to the idea that positive ethnic identity can serve as a protective factor among vulnerable minoritized groups in the U.S. Promotion of a positive ethnic identity, with a particular focus on ethnic private regard, may serve as an effective intervention protecting immigrant youth from negative outcomes due to xenophobic discrimination.

Future studies may expand on the results of this study by testing the impacts of different types and levels of policy on individuals—for instance, policies supportive of immigration, local- or national-level policies pertaining to immigration. Studies that employ multilevel modeling procedures can contribute to this line of research by testing differences among states or local municipalities that implement exclusionary immigration policies and those locations that do not implement such policies. The statistically significant relationship between state-level exclusionary immigration policy and self-esteem via positive ethnic identity should also be tested in other early adolescent immigrant groups. Though Latinx groups form a large percentage of the immigrant population in the U.S., it is important to understand the effects of immigration policies on other groups, as anti-immigrant sentiment can extend to all immigrants even if a policy targets a specific group.

## Conclusion

Studies have previously demonstrated the effects of exclusionary immigration policy on mental health, as well as the effects of ethnic identity on youth self-esteem, among Latinx early adolescents. Yet no recent studies have demonstrated the mediating relations among these variables. Analyses from this study showed that the effects of SB 1070 on later self-esteem among Latinx youth were mediated by ethnic private regard, as well as by all three ethnic identity dimensions combined. Tests of the specific indirect effects of SB 1070 on later self-esteem through ethnic centrality and ethnic public regard were not significant. These results lend support to the RIM by demonstrating longitudinal links among experiences of discrimination, positive affect regarding one’s ethnic identity, and psychological well-being (measured as self-esteem in this study). This study’s results demonstrate the import of ethnic identity for Latinx early adolescents living in a hostile political environment where anti-immigrant sentiment is codified into government policy.

## Supplementary Information

Supplementary Material
