# Supplementary material for: A Longitudinal Mediation Analysis of the Interrelations among Exclusionary Immigration Policy, Ethnic Identity, and Self-Esteem of Latinx Early Adolescents
Source: J Youth Adolesc. 2023 Jun 16;52(10):2045–60. doi: 10.1007/s10964-023-01801-x (PMC10371921; doi:10.1007/s10964-023-01801-x)
Supplement: Supplementary file 1 — Supplementary Material [file 10964_2023_1801_MOESM1_ESM.docx]

**Article Title:** A Longitudinal Mediation Analysis of the Interrelations among Exclusionary Immigration Policy, Ethnic Identity, and Self-Esteem of Latinx Early Adolescents

**Journal Name:** Journal of Youth and Adolescence

**Author Names:** Madonna P. Cadiz, LCSW; Carlos Santos, PhD; Tristan D. Tibbe

**Affiliation and Email Address of the Corresponding Author:** UCLA; [madonna.cadiz@gmail.com](mailto:madonna.cadiz@gmail.com)

**Supplementary Material**

**Factor Analyses for *Affected by SB 1070* Measure**

To increase confidence in the psychometric properties of the *Affected by SB 1070* measure, we subjected the items to an exploratory factor analysis (EFA) using principal axis factoring and an oblique rotation method, direct oblimin and listwise deletion (Tabachnick & Fidell, 2007). Approximately 50% (*n* = 448) of the complete cases in our sample were randomly selected for the EFA, and the Kaiser-Meyer-Olkin (KMO) value was 0.83, which is well above the recommended minimum of 0.6 to demonstrate sampling adequacy. Bartlett’s test of sphericity resulted in a value of 𝑋^2^ = 951.84 (*p* < .001), indicating the data were appropriate for factor analyses. Results from this EFA indicated a clear one factor structure, with factor loadings ranging from .80 to .90, and with the single factor accounting for 80.58% of the variance. Communality values ranged from .64 to .81, indicating the one-factor solution adequately explains enough of the total variation found in the data (Child, 2006). We then conducted confirmatory factor analysis (CFA) with the remaining half of the sample (*n* = 443), with a KMO value of 0.82 and a Bartlett’s test of sphericity value of 𝑋^2^ = 863.30 (*p* < .001) indicating the data were conducive to factor analysis. Results showed that the one-factor solution accounted for 81.84% of the variance. With factor loadings ranging from .84 to .92 and communality values ranging from .71 to .84, results from the CFA confirm the one-factor structure of the SB 1070 measure.

**Sensitivity Analysis 1 (Demographic Variables Excluded)**

***Missing Data Rates***

|  | Affected by SB 1070 | Self-Esteem | Ethnic Centrality | Ethnic Private Regard | Ethnic Public Regard |
| --- | --- | --- | --- | --- | --- |
| Auxiliary Var | NA | 42% | 42% | 42% | 42% |
| Wave 1 | 34% | 29% | 28% | 28% | 28% |
| Wave 2 | NA | 38% | 38% | 38% | 38% |

The following missing data percentages were observed in the variables included in the analysis model (analysis variables) and the auxiliary variables used in the imputation procedure:

***Imputation/Analysis Procedure Details***

Model-based multiple imputation (Enders, 2022) for the final analyses of this project was conducted using Blimp Version 3.0.52 (Keller & Enders, 2021). Blimp was chosen over popular multiple imputation packages in R such as Amelia (Honaker et al., 2011) and mice (van Buuren et al., 2011) because of the additional modelling flexibilities afforded by its imputation algorithms (see Enders, 2022; Keller & Enders, 2021; see the Sensitivity Analysis subsection for a comparison of these multiple imputation programs). The Blimp script used to carry out the final imputation procedure is available at <https://osf.io/sgx2p/>. Measurements for all three mediators (ethnic centrality, ethnic private regard, and ethnic public regard) and the outcome variable (self-esteem) that were taken prior to wave 1 were used as auxiliary variables in the imputation procedure. This was done because measurements taken at earlier timepoints can offer information about missing measurements at later timepoints in multiple imputation for longitudinal data (e.g., see Raykov & West, 2016), thus making the missing at random (MAR) assumption (Little & Rubin, 1987; Rubin, 1976) more plausible.

The partially factored specification (Keller & Enders, 2021) was used to impute analysis variables (i.e., those involved in the actual mediation analysis), with the imputation models mirroring the regression models used to conduct the mediation analysis. The auxiliary variables were imputed using sequential specification as described in Keller and Enders (2021). Preliminary runs of the imputation procedure revealed that the distributions of the affected by SB 1070 variable and the three ethnic private regard variables (i.e., the wave 1, wave 2, and auxiliary variables) were too skewed for the imputation procedure to produce imputed values that matched their distributions, and thus a Yeo-Johnson (Yeo & Johnson, 2000) transformation was applied to all four variables. These variables were also median-centered in the imputation procedure to improve convergence as described in Keller and Enders (2021). All iterations of the Blimp script, as well as the R script used to examine/compare the distributions of their imputations, are available at <https://osf.io/sgx2p/>.

For the final run of the Blimp multiple imputation procedure, a burn-in period of 5000 iterations was used, as this allowed the potential scale reduction factor (Gelman & Rubin, 1992) to have adequate iterations to drop below 1.05, indicating acceptable convergence (Keller & Enders, 2021). An additional 10,000 iterations spread across 100 MCMC chains were then run to generate a total of 100 imputed datasets that were saved for analysis in R Version 4.0.2 (R Core Team, 2021). The R code used to conduct the final analysis of the data is available at <https://osf.io/sgx2p/>. To obtain point estimates of the specific and total indirect effects, the necessary regression analyses were conducted in each complete imputed data set and then the indirect effects were calculated for each one. These indirect effects were finally pooled according to Rubin’s (1987) Rules by averaging the indirect effect estimates across imputed datasets (e.g., see Enders, 2022; Zhang & Wang, 2013).

To conduct inference on these indirect effects, the imputation-then-bootstrap procedure introduced by Wu and Jia (2013) was used. In their method, missing data are multiply imputed first, and then bootstrapping occurs within each complete imputed dataset. This imputation-then-bootstrap approach was chosen over Zhang and Wang’s (2013) bootstrap-then-imputation approach because it has not been shown to perform any worse than the latter method when used with the indirect effect and it is more computationally efficient (Wu & Jia, 2013; see the Sensitivity Analysis subsection for a comparison of these approaches).

Thus, following the procedure of Wu and Jia (2013), we drew 1000 bootstrap samples from each of our 100 imputed datasets and calculated the bootstrap indirect effects in each. We then pooled these 100 X 1000 = 100,000 bootstrap estimates to form one sampling distribution for each indirect effect of interest. A bootstrap confidence interval was formed for each indirect effect using these distributions. We used the percentile bootstrap confidence interval because the alternative bias-corrected bootstrap confidence interval has been shown to have inflated type I error rates in certain situations (Biesanz et al., 2010; Chen & Fritz, 2021; Fritz et al., 2012). Pooled bootstrap standard error estimates were also computed for the indirect effects by applying the variance equation described in Schomaker and Heumann (2018) to the variance of the bootstrap distribution generated in each imputed dataset and then taking its square root. A .csv file containing all the output from the final analysis is available at <https://osf.io/sgx2p/>.

***Sensitivity Analysis***

Deciding which methods and models to use in our final analysis involved making a series of decisions that could potentially have impacted our results. Thus, we examined how changing those decisions would change our results through a sensitivity analysis. First, the final mediation analysis used in our project was based on multiple imputation conducted in Blimp, but popular alternative programs including the Amelia and mice R packages could have been used instead. Also, the Wu and Jia (2013) imputation-then-bootstrap approach was implemented instead of the bootstrap-then-imputation approach of Zhang and Wang (2013). Finally, the parallel mediation model we based our final analysis on included wave 1 measurements of all three mediators and the outcome variable in all regression equations to control for prior levels of these variables (similar to the ANCOVA model discussed in Valente & MacKinnon, 2017). The following four regression equations constitute this final model:

|  | $\hat{M}_{1i}$ = *i_M1_* + *a_1_X*_𝑖_ + *u_1_U_1_*_𝑖_ + *u_2_U_2_*_𝑖_ + *u_3_U_3_*_𝑖_ + *u_4_U_4_*_𝑖_ | (1) |
| --- | --- | --- |
|  | $\hat{M}_{2i}$ = *i_M2_* + *a_2_X*_𝑖_ + *u_1_U_1_*_𝑖_ + *u_2_U_2_*_𝑖_ + *u_3_U_3_*_𝑖_ + *u_4_U_4_*_𝑖_ | (2) |
|  | $\hat{M}_{3i}$ = *i_M3_* + *a_3_X*_𝑖_ + *u_1_U_1_*_𝑖_ + *u_2_U_2_*_𝑖_ + *u_3_U_3_*_𝑖_ + *u_4_U_4_*_𝑖_ | (3) |
|  | *Ŷ*_𝑖_ = *i_Y_* + *c’X*_𝑖_ + *b_1_M*_1𝑖_ + *b_2_M*_2𝑖_ + *b_3_M*_3𝑖_ + *u_1_U_1_*_𝑖_ + *u_2_U_2_*_𝑖_ + *u_3_U_3_*_𝑖_ + *u_4_U_4_*_𝑖_ | (4) |

In the above equations, the uppercase letters represent variables, the lowercase letters appearing next to the uppercase letters represent regression coefficients, and the *i* terms represent the intercept for each equation. The *i*s that appear in the subscripts of the variables indicate that these are the variable values for observation *i*, and the hat notation above the variable that appears on the left side of each equation indicates that it is the estimated value of that variable generated by the regression line. Equation 1 is the equation for wave 2 ethnic centrality (*M*_1_), Equation 2 is for wave 2 ethnic private regard (*M*_2_), Equation 3 is for ethnic public regard (*M*_3_), and Equation 4 is for wave 2 self-esteem (*Y*). wave 1 ethnic centrality, wave 1 ethnic private regard, wave 1 ethnic public regard, and wave 1 self-esteem are represented in the equations by *U_1_*, *U_2_*, *U_3_*, and *U_4_*, respectively. Note that *U_1_*, *U_2_*, *U_3_*, and *U_4_* all appear in all four equations above. Alternatively, we could have included in each equation only the wave 1 variable that corresponded to the outcome variable of that equation. This model is represented by the following four equations:

| $\hat{M}_{1i}$ = *i_M1_* + *a_1_X*_𝑖_ + *u_1_U_1_*_𝑖_ | (5) |
| --- | --- |
| $\hat{M}_{2i}$ = *i_M2_* + *a_2_X*_𝑖_ + *u_2_U_2_*_𝑖_ | (6) |
| $\hat{M}_{3i}$ = *i_M3_* + *a_3_X*_𝑖_ + *u_3_U_3_*_𝑖_ | (7) |
| *Ŷ*_𝑖_ = *i_Y_* + *c’X*_𝑖_ + *b_1_M*_1𝑖_ + *b_2_M*_2𝑖_ + *b_3_M*_3𝑖_ + *u_4_U_4_*_𝑖_ | (8) |

Here, Equation 5 contains only wave 1 ethnic centrality (*U_1_*) because wave 2 ethnic centrality (*M*_1_) is the outcome variable. Similarly, Equation 6 contains *U*_2_ and *M*_2_, Equation 7 contains *U*_3_ and *M*_3_, and Equation 8 contains *U*_4_ and *Y*. Yet another alternative model we considered combined Equations 5, 6, and 7 from the first alternative model with Equation 4 from our final model, thus only including all wave 1 covariates in the equation for wave 2 self-esteem because it is the only equation in which all wave 2 variables appear.

The two alternative imputation programs (Amelia and mice), two possible impute/bootstrap procedures (imputation-then-bootstrap and bootstrap-then-imputation), and three analysis models (all wave 1 variables in all equations, a single wave 1 variable in each equation, and all wave 1 variables in the equation for *Y* with a single wave 1 variable in every other equation) together form a total of 12 different analyses we could have used in our final paper. To see if our conclusions would have changed if we had chosen one of these alternative analyses, we ran each one and compared their results. The R code used to run this sensitivity analysis is available at <https://osf.io/sgx2p/>, as well as a .csv file containing the results of the analysis.

For comparison, Table 1 contains the specific/total indirect effect estimates and their corresponding percentile bootstrap confidence intervals we found in our final analysis using Blimp with the imputation-then-bootstrap approach and Equations 1, 2, 3, and 4 that each contain all wave 1 control variables.

**Table 1**

*Results for Final Analysis with Blimp, Imputation-then-Bootstrap, and All Wave 1 Variables in All Equations*

| Indirect Effect | Estimate | *LLCI* | *ULCI* |  |  |
| --- | --- | --- | --- | --- | --- |
| *a_1_b_1_* | 0.000 | -0.001 | 0.001 |  |  |
| *a_2_b_2_* | 0.004 | 0.001 | 0.008 |  |  |
| *a_3_b_3_* | 0.000 | -0.001 | 0.001 |  |  |
| Σ(*a_i_b_i_*) | 0.004 | 0.000 | 0.008 |  |  |

*Note.* ‘*a_1_b_1_*’ = specific indirect effect through wave 2 ethnic centrality, ‘*a_2_b_2_*’ = specific indirect effect through wave 2 ethnic private regard, ‘*a_3_b_3_*’ = specific indirect effect through wave 2 ethnic public regard, ‘Σ(*a_i_b_i_*)’ = total indirect effect, ‘*LLCI*’ = lower limit of percentile bootstrap confidence interval, ‘*ULCI*’ = upper limit of percentile bootstrap confidence interval.

By examining which percentile bootstrap confidence intervals exclude zero, we determine that the specific indirect effect through wave 2 ethnic private regard and the total indirect effect are both significantly different from zero (the lower limit of the confidence interval for the total indirect effect is 0.000376).

Tables 2, 3, 4, and 5 below contain the results for the alternative analyses we ran. Each table corresponds to a different combination of imputation program and impute/bootstrap procedure, and they all display the estimates/percentile bootstrap confidence intervals for the three different covariate patterns we considered for the analysis model.

**Table 2**

*Results for Analyses with Amelia and Bootstrap-then-Imputation*

| Covariates | Indirect Effect | Estimate | *LLCI* | *ULCI* |  |  |
| --- | --- | --- | --- | --- | --- | --- |
| **All** | *a_1_b_1_* | -0.000 | -0.002 | 0.001 |  |  |
|  | *a_2_b_2_* | 0.004 | 0.001 | 0.008 |  |  |
|  | *a_3_b_3_* | 0.000 | -0.001 | 0.001 |  |  |
|  | Σ(*a_i_b_i_*) | 0.004 | 0.001 | 0.008 |  |  |
| **Single** | *a_1_b_1_* | -0.000 | -0.002 | 0.001 |  |  |
|  | *a_2_b_2_* | 0.003 | 0.001 | 0.007 |  |  |
|  | *a_3_b_3_* | 0.000 | -0.001 | 0.001 |  |  |
|  | Σ(*a_i_b_i_*) | 0.003 | 0.000 | 0.007 |  |  |
| **Single + All** | *a_1_b_1_* | -0.000 | -0.002 | 0.002 |  |  |
|  | *a_2_b_2_* | 0.004 | 0.001 | 0.007 |  |  |
|  | *a_3_b_3_* | -0.000 | -0.001 | 0.001 |  |  |
|  | Σ(*a_i_b_i_*) | 0.004 | 0.000 | 0.008 |  |  |

*Note.* ‘**All**’ = model that contains all wave 1 covariates in all equations, ‘**Single**’ = model that contains a single wave 1 covariate in each equation, ‘**Single + All**’ = model that contains all wave 1 covariates in the equation for *Y* and a single wave 1 covariate in every other equation, ‘*a_1_b_1_*’ = specific indirect effect through wave 2 ethnic centrality, ‘*a_2_b_2_*’ = specific indirect effect through wave 2 ethnic private regard, ‘*a_3_b_3_*’ = specific indirect effect through wave 2 ethnic public regard, ‘Σ(*a_i_b_i_*)’ = total indirect effect, ‘*LLCI*’ = lower limit of percentile bootstrap confidence interval, ‘*ULCI*’ = upper limit of percentile bootstrap confidence interval.

**Table 3**

*Results for Analyses with mice and Bootstrap-then-Imputation*

| Covariates | Indirect Effect | Estimate | *LLCI* | *ULCI* |  |  |
| --- | --- | --- | --- | --- | --- | --- |
| **All** | *a_1_b_1_* | 0.000 | -0.002 | 0.001 |  |  |
|  | *a_2_b_2_* | 0.004 | 0.000 | 0.009 |  |  |
|  | *a_3_b_3_* | 0.000 | -0.001 | 0.001 |  |  |
|  | Σ(*a_i_b_i_*) | 0.004 | 0.000 | 0.008 |  |  |
| **Single** | *a_1_b_1_* | -0.000 | -0.003 | 0.000 |  |  |
|  | *a_2_b_2_* | 0.003 | 0.000 | 0.008 |  |  |
|  | *a_3_b_3_* | 0.000 | -0.001 | 0.001 |  |  |
|  | Σ(*a_i_b_i_*) | 0.003 | -0.000 | 0.006 |  |  |
| **Single + All** | *a_1_b_1_* | 0.000 | -0.002 | 0.001 |  |  |
|  | *a_2_b_2_* | 0.003 | 0.000 | 0.009 |  |  |
|  | *a_3_b_3_* | 0.000 | -0.001 | 0.001 |  |  |
|  | Σ(*a_i_b_i_*) | 0.003 | -0.000 | 0.007 |  |  |

*Note.* ‘**All**’ = model that contains all wave 1 covariates in all equations, ‘**Single**’ = model that contains a single wave 1 covariate in each equation, ‘**Single + All**’ = model that contains all wave 1 covariates in the equation for *Y* and a single wave 1 covariate in every other equation, ‘*a_1_b_1_*’ = specific indirect effect through wave 2 ethnic centrality, ‘*a_2_b_2_*’ = specific indirect effect through wave 2 ethnic private regard, ‘*a_3_b_3_*’ = specific indirect effect through wave 2 ethnic public regard, ‘Σ(*a_i_b_i_*)’ = total indirect effect, ‘*LLCI*’ = lower limit of percentile bootstrap confidence interval, ‘*ULCI*’ = upper limit of percentile bootstrap confidence interval.

**Table 4**

*Results for Analyses with Amelia and Imputation-then-Bootstrap*

| Covariates | Indirect Effect | Estimate | *LLCI* | *ULCI* |  |  |
| --- | --- | --- | --- | --- | --- | --- |
| **All** | *a_1_b_1_* | 0.000 | -0.001 | 0.001 |  |  |
|  | *a_2_b_2_* | 0.004 | 0.001 | 0.008 |  |  |
|  | *a_3_b_3_* | 0.000 | -0.001 | 0.001 |  |  |
|  | Σ(*a_i_b_i_*) | 0.004 | 0.001 | 0.008 |  |  |
| **Single** | *a_1_b_1_* | -0.000 | -0.002 | 0.001 |  |  |
|  | *a_2_b_2_* | 0.003 | 0.001 | 0.007 |  |  |
|  | *a_3_b_3_* | 0.000 | -0.001 | 0.001 |  |  |
|  | Σ(*a_i_b_i_*) | 0.003 | 0.000 | 0.007 |  |  |
| **Single + All** | *a_1_b_1_* | 0.000 | -0.001 | 0.002 |  |  |
|  | *a_2_b_2_* | 0.004 | 0.001 | 0.008 |  |  |
|  | *a_3_b_3_* | 0.000 | -0.001 | 0.001 |  |  |
|  | Σ(*a_i_b_i_*) | 0.004 | 0.001 | 0.008 |  |  |

*Note.* ‘**All**’ = model that contains all wave 1 covariates in all equations, ‘**Single**’ = model that contains a single wave 1 covariate in each equation, ‘**Single + All**’ = model that contains all wave 1 covariates in the equation for *Y* and a single wave 1 covariate in every other equation, ‘*a_1_b_1_*’ = specific indirect effect through wave 2 ethnic centrality, ‘*a_2_b_2_*’ = specific indirect effect through wave 2 ethnic private regard, ‘*a_3_b_3_*’ = specific indirect effect through wave 2 ethnic public regard, ‘Σ(*a_i_b_i_*)’ = total indirect effect, ‘*LLCI*’ = lower limit of percentile bootstrap confidence interval, ‘*ULCI*’ = upper limit of percentile bootstrap confidence interval.

**Table 5**

*Results for Analyses with mice and Imputation-then-Bootstrap*

| Covariates | Indirect Effect | Estimate | *LLCI* | *ULCI* |  |  |
| --- | --- | --- | --- | --- | --- | --- |
| **All** | *a_1_b_1_* | -0.000 | -0.001 | 0.001 |  |  |
|  | *a_2_b_2_* | 0.004 | 0.001 | 0.008 |  |  |
|  | *a_3_b_3_* | 0.000 | -0.001 | 0.001 |  |  |
|  | Σ(*a_i_b_i_*) | 0.004 | 0.001 | 0.008 |  |  |
| **Single** | *a_1_b_1_* | -0.000 | -0.002 | 0.001 |  |  |
|  | *a_2_b_2_* | 0.003 | 0.000 | 0.007 |  |  |
|  | *a_3_b_3_* | 0.000 | -0.001 | 0.001 |  |  |
|  | Σ(*a_i_b_i_*) | 0.003 | 0.000 | 0.007 |  |  |
| **Single + All** | *a_1_b_1_* | -0.000 | -0.002 | 0.001 |  |  |
|  | *a_2_b_2_* | 0.003 | 0.001 | 0.007 |  |  |
|  | *a_3_b_3_* | 0.000 | -0.001 | 0.001 |  |  |
|  | Σ(*a_i_b_i_*) | 0.003 | 0.000 | 0.007 |  |  |

*Note.* ‘**All**’ = model that contains all wave 1 covariates in all equations, ‘**Single**’ = model that contains a single wave 1 covariate in each equation, ‘**Single + All**’ = model that contains all wave 1 covariates in the equation for *Y* and a single wave 1 covariate in every other equation, ‘*a_1_b_1_*’ = specific indirect effect through wave 2 ethnic centrality, ‘*a_2_b_2_*’ = specific indirect effect through wave 2 ethnic private regard, ‘*a_3_b_3_*’ = specific indirect effect through wave 2 ethnic public regard, ‘Σ(*a_i_b_i_*)’ = total indirect effect, ‘*LLCI*’ = lower limit of percentile bootstrap confidence interval, ‘*ULCI*’ = upper limit of percentile bootstrap confidence interval.

Examining Tables 2, 3, 4, and 5, we see that the only two instances where the conclusions reached differ from the conclusions reached in our final analysis occur in Table 3: When conducting multiple imputation using mice with the bootstrap-then-imputation procedure and the model that contains either a single wave 1 variable in each equation or all wave 1 variables in the equation for *Y* with a single wave 1 variable in every other equation, the total indirect effect is not significantly different from zero (i.e., the confidence intervals for the total indirect effects in these models contain zero). The conclusions reached about the specific indirect effects, on the other hand, are always the same regardless of the analysis conducted: The specific indirect effect through wave 2 ethnic private regard is always found to significantly differ from zero, and the other two specific indirect effects are never significant.

**Sensitivity Analysis 2 (Demographic Variables Included)**

***Missing Data Rates***

|  | Affected by SB 1070 | Self-Esteem | Ethnic Centrality | Ethnic Private Regard | Ethnic Public Regard | Age | Gender | Immigrant Generation |
| --- | --- | --- | --- | --- | --- | --- | --- | --- |
| Auxiliary Var | NA | 42% | 42% | 42% | 42% | 42% | 42% | 38% |
| Wave 1 | 34% | 29% | 28% | 28% | 28% | 28% | 28% | 28% |
| Wave 2 | NA | 38% | 38% | 38% | 38% | NA | NA | NA |

The following missing data percentages were observed in the variables included in the analysis model (analysis variables) and the auxiliary variables used in the imputation procedure for the second sensitivity analysis:

***Imputation/Analysis Procedure Details***

The exact same model-based multiple imputation procedure implemented in Blimp that was used for the original imputation of this paper was used here as well, with the following exceptions: age, gender, and immigrant generation measurements taken at wave 1 were added to the main analysis models, and measurements taken on all three variables prior to wave 1 were used as auxiliary variables. The dichotomous gender and multicategorical immigrant generation variables were treated as ordinal variables in Blimp to account for their discrete nature. Also, the most up-to-date version of Blimp (Version 3.0.66; Keller & Enders, 2022) was used. The Blimp script used to carry out this imputation procedure is available at <https://osf.io/sgx2p/>.

For the final run of this Blimp multiple imputation procedure, a burn-in period of 5000 iterations was once again used, and an additional 10,000 iterations spread across 100 MCMC chains were again run to generate a total of 100 imputed datasets that were saved for analysis in R Version 4.1.1 (R Core Team, 2021). The 5000-iteration burn in was sufficient for the potential scale reduction factor (PSR) to drop below the 1.05 threshold for all parameters except those involved in the model for the gender auxiliary variable, which had a maximum PSR of 1.226 at the end of the burn-in period.

The R code used to conduct the analysis of this imputed data is available at <https://osf.io/sgx2p/>. The same procedures used for the original final analysis of this paper were once again used here, except that the age, gender, and immigrant generation wave 1 measurements were added as covariates to all analysis models. See Table 6 for the indirect effect results. Also, a .csv file containing all the output from this analysis is available at <https://osf.io/sgx2p/>.

***Sensitivity Analysis***

To see how sensitive the results in Table 6 were to changes in methods and models, the exact same factors altered in the first sensitivity analysis were again altered here. The only exception: age, gender, and immigrant generation variables were added to the imputation procedures and the final analyses. During imputation with Amelia, gender and immigrant generation were considered ordinal variables as discussed in Honaker et al. (2011), and measurements of age and immigrant generation taken prior to wave 1 were used as auxiliary variables. The prior measurement of gender was dropped from the imputation procedure because its high correlation with wave 1 gender prevented the procedure from completing successfully. During imputation with mice, prior measurements of age and immigrant generation were once again used as auxiliary variables. All variables were imputed with predictive mean matching as described in van Buuren et al. (2011). In the analyses, wave 1 age, wave 1 gender, and two immigrant generation dummy variables (“Child and Both Parents Foreign Born” served as the reference group) were added to each model that was fit. Thus, these four variables were added to Equations 1 through 8, resulting in an additional four regression coefficients estimated for each equation. For example, Equations 1 through 4 were modified to become:

|  | $\hat{M}_{1i}$ = *i_M1_* + *a_1_X*_𝑖_ + *u_1_U_1_*_𝑖_ + *u_2_U_2_*_𝑖_ + *u_3_U_3_*_𝑖_ + *u_4_U_4_*_𝑖_ + *u_5_U_5_*_𝑖_ + *u_6_U_6_*_𝑖_ + *u_7_U_7_*_𝑖_ + *u_8_U_8_*_𝑖_ | (9) |
| --- | --- | --- |
|  | $\hat{M}_{2i}$ = *i_M2_* + *a_2_X*_𝑖_ + *u_1_U_1_*_𝑖_ + *u_2_U_2_*_𝑖_ + *u_3_U_3_*_𝑖_ + *u_4_U_4_*_𝑖_ + *u_5_U_5_*_𝑖_ + *u_6_U_6_*_𝑖_ + *u_7_U_7_*_𝑖_ + *u_8_U_8_*_𝑖_ | (10) |
|  | $\hat{M}_{3i}$ = *i_M3_* + *a_3_X*_𝑖_ + *u_1_U_1_*_𝑖_ + *u_2_U_2_*_𝑖_ + *u_3_U_3_*_𝑖_ + *u_4_U_4_*_𝑖_ + *u_5_U_5_*_𝑖_ + *u_6_U_6_*_𝑖_ + *u_7_U_7_*_𝑖_ + *u_8_U_8_*_𝑖_ | (11) |
|  | *Ŷ*_𝑖_ = *i_Y_* + *c’X*_𝑖_ + *b_1_M*_1𝑖_ + *b_2_M*_2𝑖_ + *b_3_M*_3𝑖_ + *u_1_U_1_*_𝑖_ + *u_2_U_2_*_𝑖_ + *u_3_U_3_*_𝑖_ + *u_4_U_4_*_𝑖_ + *u_5_U_5_*_𝑖_ + *u_6_U_6_*_𝑖_ + *u_7_U_7_*_𝑖_ + *u_8_U_8_*_𝑖_ | (12) |

Note that the only difference between Equations 1 through 4 and Equations 9 through 12 is the addition of *U_5_* (wave 1 age), *U_6_* (wave 1 gender), and *U_7_* and *U_8_* (the two immigrant generation dummy variables). The R code used to run this second sensitivity analysis is available at <https://osf.io/sgx2p/>, as well as a .csv file containing the results.

Again, for comparison, Table 6 contains the specific/total indirect effect estimates and their corresponding percentile bootstrap confidence intervals found in our analysis using Blimp with wave 1 age, wave 1 gender, and the two immigrant generation dummy variables added to all equations.

**Table 6**

*Results for Analysis with Blimp, Imputation-then-Bootstrap, All Wave 1 Variables in All Equations (including Age and Gender), and Two Immigrant Generation Dummy Variables in All Equations*

| Indirect Effect | Estimate | *LLCI* | *ULCI* |  |  |
| --- | --- | --- | --- | --- | --- |
| *a_1_b_1_* | 0.000 | -0.001 | 0.001 |  |  |
| *a_2_b_2_* | 0.003 | 0.000 | 0.008 |  |  |
| *a_3_b_3_* | 0.000 | -0.001 | 0.001 |  |  |
| Σ(*a_i_b_i_*) | 0.003 | -0.000 | 0.008 |  |  |

*Note.* ‘*a_1_b_1_*’ = specific indirect effect through wave 2 ethnic centrality, ‘*a_2_b_2_*’ = specific indirect effect through wave 2 ethnic private regard, ‘*a_3_b_3_*’ = specific indirect effect through wave 2 ethnic public regard, ‘Σ(*a_i_b_i_*)’ = total indirect effect, ‘*LLCI*’ = lower limit of percentile bootstrap confidence interval, ‘*ULCI*’ = upper limit of percentile bootstrap confidence interval.

By examining which percentile bootstrap confidence intervals exclude zero, we determine that the specific indirect effect through wave 2 ethnic private regard is significantly different from zero (the lower limit of its confidence interval is 0.00007).

Tables 7, 8, 9, and 10 below contain the results for the alternative analyses we ran. Each table corresponds to a different combination of imputation program and impute/bootstrap procedure, and they all display the estimates/percentile bootstrap confidence intervals for the three different covariate patterns we considered for the analysis model (see the Sensitivity Analysis 1 section for more details).

**Table 7**

*Results for Analyses with Amelia and Bootstrap-then-Imputation*

| Covariates | Indirect Effect | Estimate | *LLCI* | *ULCI* |  |  |
| --- | --- | --- | --- | --- | --- | --- |
| **All** | *a_1_b_1_* | -0.000 | -0.002 | 0.001 |  |  |
|  | *a_2_b_2_* | 0.004 | 0.000 | 0.007 |  |  |
|  | *a_3_b_3_* | 0.000 | -0.001 | 0.001 |  |  |
|  | Σ(*a_i_b_i_*) | 0.003 | -0.000 | 0.007 |  |  |
| **Single** | *a_1_b_1_* | -0.000 | -0.002 | 0.001 |  |  |
|  | *a_2_b_2_* | 0.004 | 0.001 | 0.008 |  |  |
|  | *a_3_b_3_* | 0.000 | -0.001 | 0.001 |  |  |
|  | Σ(*a_i_b_i_*) | 0.004 | 0.000 | 0.008 |  |  |
| **Single + All** | *a_1_b_1_* | -0.000 | -0.002 | 0.001 |  |  |
|  | *a_2_b_2_* | 0.004 | 0.000 | 0.007 |  |  |
|  | *a_3_b_3_* | 0.000 | -0.001 | 0.001 |  |  |
|  | Σ(*a_i_b_i_*) | 0.004 | 0.000 | 0.007 |  |  |

*Note.* ‘**All**’ = model that contains all wave 1 covariates in all equations (including age, gender, and immigrant generation dummy variables), ‘**Single**’ = model that contains a single wave 1 covariate in each equation (and wave 1 age, wave 1 gender, and immigrant generation dummy variables in all equations), ‘**Single + All**’ = model that contains all wave 1 covariates in the equation for *Y* and a single wave 1 covariate in every other equation (and wave 1 age, wave 1 gender, and immigrant generation dummy variables in all equations), ‘*a_1_b_1_*’ = specific indirect effect through wave 2 ethnic centrality, ‘*a_2_b_2_*’ = specific indirect effect through wave 2 ethnic private regard, ‘*a_3_b_3_*’ = specific indirect effect through wave 2 ethnic public regard, ‘Σ(*a_i_b_i_*)’ = total indirect effect, ‘*LLCI*’ = lower limit of percentile bootstrap confidence interval, ‘*ULCI*’ = upper limit of percentile bootstrap confidence interval.

**Table 8**

*Results for Analyses with mice and Bootstrap-then-Imputation*

| Covariates | Indirect Effect | Estimate | *LLCI* | *ULCI* |  |  |
| --- | --- | --- | --- | --- | --- | --- |
| **All** | *a_1_b_1_* | -0.000 | -0.002 | 0.001 |  |  |
|  | *a_2_b_2_* | 0.003 | 0.001 | 0.006 |  |  |
|  | *a_3_b_3_* | 0.000 | -0.001 | 0.001 |  |  |
|  | Σ(*a_i_b_i_*) | 0.003 | -0.000 | 0.006 |  |  |
| **Single** | *a_1_b_1_* | 0.000 | -0.001 | 0.001 |  |  |
|  | *a_2_b_2_* | 0.003 | 0.001 | 0.007 |  |  |
|  | *a_3_b_3_* | 0.000 | -0.001 | 0.001 |  |  |
|  | Σ(*a_i_b_i_*) | 0.003 | 0.000 | 0.007 |  |  |
| **Single + All** | *a_1_b_1_* | 0.000 | -0.002 | 0.001 |  |  |
|  | *a_2_b_2_* | 0.003 | 0.001 | 0.007 |  |  |
|  | *a_3_b_3_* | 0.000 | -0.001 | 0.001 |  |  |
|  | Σ(*a_i_b_i_*) | 0.003 | -0.000 | 0.007 |  |  |

*Note.* ‘**All**’ = model that contains all wave 1 covariates in all equations (including age, gender, and immigrant generation dummy variables), ‘**Single**’ = model that contains a single wave 1 covariate in each equation (and wave 1 age, wave 1 gender, and immigrant generation dummy variables in all equations), ‘**Single + All**’ = model that contains all wave 1 covariates in the equation for *Y* and a single wave 1 covariate in every other equation (and wave 1 age, wave 1 gender, and immigrant generation dummy variables in all equations), ‘*a_1_b_1_*’ = specific indirect effect through wave 2 ethnic centrality, ‘*a_2_b_2_*’ = specific indirect effect through wave 2 ethnic private regard, ‘*a_3_b_3_*’ = specific indirect effect through wave 2 ethnic public regard, ‘Σ(*a_i_b_i_*)’ = total indirect effect, ‘*LLCI*’ = lower limit of percentile bootstrap confidence interval, ‘*ULCI*’ = upper limit of percentile bootstrap confidence interval.

**Table 9**

*Results for Analyses with Amelia and Imputation-then-Bootstrap*

| Covariates | Indirect Effect | Estimate | *LLCI* | *ULCI* |  |  |
| --- | --- | --- | --- | --- | --- | --- |
| **All** | *a_1_b_1_* | 0.000 | -0.002 | 0.001 |  |  |
|  | *a_2_b_2_* | 0.003 | 0.001 | 0.007 |  |  |
|  | *a_3_b_3_* | 0.000 | -0.001 | 0.001 |  |  |
|  | Σ(*a_i_b_i_*) | 0.003 | 0.000 | 0.007 |  |  |
| **Single** | *a_1_b_1_* | -0.000 | -0.002 | 0.001 |  |  |
|  | *a_2_b_2_* | 0.004 | 0.001 | 0.008 |  |  |
|  | *a_3_b_3_* | 0.000 | -0.001 | 0.001 |  |  |
|  | Σ(*a_i_b_i_*) | 0.004 | 0.001 | 0.008 |  |  |
| **Single + All** | *a_1_b_1_* | -0.000 | -0.002 | 0.001 |  |  |
|  | *a_2_b_2_* | 0.004 | 0.001 | 0.007 |  |  |
|  | *a_3_b_3_* | 0.000 | -0.001 | 0.001 |  |  |
|  | Σ(*a_i_b_i_*) | 0.004 | 0.000 | 0.008 |  |  |

*Note.* ‘**All**’ = model that contains all wave 1 covariates in all equations (including age, gender, and immigrant generation dummy variables), ‘**Single**’ = model that contains a single wave 1 covariate in each equation (and wave 1 age, wave 1 gender, and immigrant generation dummy variables in all equations), ‘**Single + All**’ = model that contains all wave 1 covariates in the equation for *Y* and a single wave 1 covariate in every other equation (and wave 1 age, wave 1 gender, and immigrant generation dummy variables in all equations), ‘*a_1_b_1_*’ = specific indirect effect through wave 2 ethnic centrality, ‘*a_2_b_2_*’ = specific indirect effect through wave 2 ethnic private regard, ‘*a_3_b_3_*’ = specific indirect effect through wave 2 ethnic public regard, ‘Σ(*a_i_b_i_*)’ = total indirect effect, ‘*LLCI*’ = lower limit of percentile bootstrap confidence interval, ‘*ULCI*’ = upper limit of percentile bootstrap confidence interval.

**Table 10**

*Results for Analyses with mice and Imputation-then-Bootstrap*

| Covariates | Indirect Effect | Estimate | *LLCI* | *ULCI* |  |  |
| --- | --- | --- | --- | --- | --- | --- |
| **All** | *a_1_b_1_* | -0.000 | -0.002 | 0.001 |  |  |
|  | *a_2_b_2_* | 0.003 | 0.000 | 0.006 |  |  |
|  | *a_3_b_3_* | 0.000 | -0.001 | 0.001 |  |  |
|  | Σ(*a_i_b_i_*) | 0.003 | -0.000 | 0.006 |  |  |
| **Single** | *a_1_b_1_* | -0.000 | -0.001 | 0.001 |  |  |
|  | *a_2_b_2_* | 0.003 | 0.000 | 0.007 |  |  |
|  | *a_3_b_3_* | 0.000 | -0.001 | 0.001 |  |  |
|  | Σ(*a_i_b_i_*) | 0.003 | 0.000 | 0.007 |  |  |
| **Single + All** | *a_1_b_1_* | -0.000 | -0.002 | 0.001 |  |  |
|  | *a_2_b_2_* | 0.003 | 0.000 | 0.007 |  |  |
|  | *a_3_b_3_* | 0.000 | -0.001 | 0.001 |  |  |
|  | Σ(*a_i_b_i_*) | 0.003 | -0.000 | 0.007 |  |  |

*Note.* ‘**All**’ = model that contains all wave 1 covariates in all equations (including age, gender, and immigrant generation dummy variables), ‘**Single**’ = model that contains a single wave 1 covariate in each equation (and wave 1 age, wave 1 gender, and immigrant generation dummy variables in all equations), ‘**Single + All**’ = model that contains all wave 1 covariates in the equation for *Y* and a single wave 1 covariate in every other equation (and wave 1 age, wave 1 gender, and immigrant generation dummy variables in all equations), ‘*a_1_b_1_*’ = specific indirect effect through wave 2 ethnic centrality, ‘*a_2_b_2_*’ = specific indirect effect through wave 2 ethnic private regard, ‘*a_3_b_3_*’ = specific indirect effect through wave 2 ethnic public regard, ‘Σ(*a_i_b_i_*)’ = total indirect effect, ‘*LLCI*’ = lower limit of percentile bootstrap confidence interval, ‘*ULCI*’ = upper limit of percentile bootstrap confidence interval.

Examining Tables 7, 8, 9, and 10, we see that the only instances where the conclusions reached differ from the conclusions reached in our analysis from Table 6 occur with the total indirect effect: Five times in Tables 7 through 10, the total indirect effect is not significantly different from zero (indicated by a confidence interval that contains zero), but the other seven times it is significantly different from zero (indicated by a confidence interval that excludes zero). The conclusions reached about the specific indirect effects, on the other hand, are always the same regardless of the analysis conducted: The specific indirect effect through wave 2 ethnic private regard is always found to significantly differ from zero, and the other two specific indirect effects are never significant.

**References**

Biesanz, J. C., Falk, C. F., & Savalei, V. (2010). Assessing mediational models: Testing and interval estimation for indirect effects. *Multivariate Behavioral Research, 45*, 661–701.

Chen, D., & Fritz, M. S. (2021). Comparing alternative corrections for bias in the bias-corrected bootstrap test of mediation. *Evaluation and the Health Professions, 44*(4), 416–427.

Child, D. (2006). *The essentials of factor analysis* (3rd ed.). New York, NY: Continuum

International Publishing Group.

Enders, C. K. (2022). *Applied missing data analysis 2.0*. Guilford Press.

Fritz, M. S., Taylor, A. B., & MacKinnon, D. P. (2012). Explanation of two anomalousresults in statistical mediation analysis. *Multivariate Behavioral Research, 47*(1), 61–87.

Gelman, A., & Rubin, D. B. (1992). Inference from iterative simulation using multiple

sequences. *Statistical Science, 7*, 457–472. doi:10.1214/ss/1177011136

Honaker, J., King, G., & Blackwell, M. (2011). Amelia II: A program for missing data. *Journal of Statistical Software*, *45*(7), 1–47.

Keller, B. T., & Enders, C. K. (2022). *Blimp user’s guide (Version 3)*. Retrieved from

[www.appliedmissingdata.com/multilevel-imputation.html](http://www.appliedmissingdata.com/multilevel-imputation.html)

Little, R. J. A., & Rubin, D. B. (1987). *Statistical analysis with missing data (1st)*. New York: Wiley.

R Core Team (2021). *R: A language and environment for statistical computing*. R Foundation for Statistical Computing. Vienna, Austria. Retrieved from <https://www.R-project.org/>

Raykov, T., & West, B. T. (2016). On enhancing plausibility of the missing at random assumption in incomplete data analyses via evaluation of response-auxiliary variable correlations*. Structural Equation Modeling: A Multidisciplinary Journal, 23*(1), 45–53. DOI: 10.1080/10705511.2014.937848

Rubin, D. B. (1976). Inference and missing data. *Biometrika, 63*, 581–592.

Rubin, D. B. (1987). *Multiple imputation for nonresponse in surveys*. New York: Wiley.

Schomaker, M., & Heumann, C. (2018). Bootstrap inference when using multiple imputation. *Statistics in Medicine*, *37*(14), 2252–2266.

Tabachnick, B. G., & Fidell, L. S. (2007). *Using multivariate statistics* (7th Edition). Pearson.

https://www.pearsonhighered.com/assets/preface/0/1/3/4/0134790545.pdf

Valente, M. J., & MacKinnon, D. P. (2017). Comparing models of change to estimate the mediated effect in the pretest-posttest control group design. *Structural Equation Modeling: A Multidisciplinary Journal*, *24*(3), 428–450.

van Buuren, S., & Groothuis–Oudshoorn, K. (2011). MICE: Multivariate imputation by

chained equations in R. *Journal of Statistical Software, 45*(3), 1–67.

Wu, W., & Jia, F. (2013). A new procedure to test mediation with missing data through nonparametric bootstrapping and multiple imputation. *Multivariate Behavioral Research, 48*(5), 663–691.

Yeo, I. K., & Johnson, R. A. (2000). A new family of power transformations to improve

normality or symmetry. *Biometrika, 87*(4), 954–959.

Zhang, Z., & Wang, L. (2013). Methods for mediation analysis with missing data. *Psychometrika, 78*(1), 154–184.
